# Paramedics and health promotion

**DOI:** 10.1177/17579139211053363

**Published:** 2021-10-21

**Authors:** B Schofield, S McClean

**Affiliations:** Senior Research Fellow, School of Health and Wellbeing, University of West of England, Glenside Campus, Bristol BS16 1DD, UK; Associate Professor of Public Health, School of Health and Wellbeing, University of West of England, Bristol, UK

*In this opinion piece, Schofield and McClean report on the rationale for the potential for a change in scope of practice for paramedics to provide health promotion. The authors outline how this needs to be supported by further research to support the acceptability of this expansion to their traditional role both by the profession and by patients*.

**Figure:**
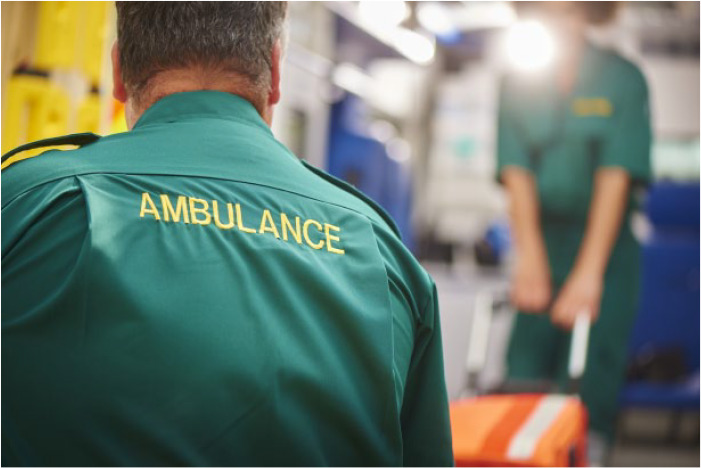


The NHS continues to face rises in the demand for urgent and emergency care. Paramedics provide out-of-hospital urgent and emergency care, often in the most difficult and unpredictable circumstances. There is a need for more ambulances and more personnel to staff these units. The College of Paramedics describes paramedics as ‘registered healthcare professionals who have a unique role that crosses healthcare, public health, social care and public safety, they work autonomously providing care in a range of situations’.^1^ In the UK, paramedics are best known for working within Ambulance Services providing immediate and emergency care in response to 999 calls made by the public.

Although health promotion is in its early days in paramedicine, gradually the paramedic’s role in promoting health and wellbeing is starting to receive more attention. The College of Paramedics is including health promotion as part of the scope of practice.^2^ The ambulance services are acknowledging the importance of using patient contact time with patients for this purpose.^3^ Public Health England, NHS England and Health Education England have produced a consensus statement describing the commitment of their organisations to work together to maximise support for population behaviour change and help individuals and communities significantly reduce their risk of disease.^4^ This statement recommends the evidence-based approach to healthcare of Making Every Contact Count (MECC), which encourages all those who have contact with the public to talk about their health and wellbeing and should be applied across all health and social care organisations.^5^ Paramedics have the opportunity to support positive behavioural changes through MECC.

The Ottawa Charter, one of the cornerstones of health promotion, identifies three primary mechanisms for promoting health:

- Advocacy for health and for a holistic view of health;
- Enabling people by creating equity in terms of access, opportunities, resource availability and life skills;
- Mediation among governmental, industrial, health, community and other sectors of society which recognises the interdependent and intersectoral nature of health and wellbeing.^6^

Paramedics offer universal and equitable resource availability (by providing the same equipment and personnel to anyone calling for an ambulance at any time for any reason), are knowledgeable and they have high levels of trust among the public. They are well placed to advocate for their patients and well-positioned to mediate among and between the many agencies and organisations. Every year 10 million 999 calls are made, and 30% of these patients are treated and discharged at scene by the attending paramedic.^7^

These interactions often at the patient’s home provide a unique opportunity for paramedics to identify people with risk factors, and opportunities to provide information, brief interventions and signpost people to locally provided services.

Paramedics today fill broader roles than those encompassed within traditional models of prehospital care, in part due to ageing populations and the prevalence of chronic conditions. An expanded role may help address health workforce sustainability and shortages.

There is very limited evidence about if or how health promotion is delivered by paramedics and its acceptability to patients, their family and the profession. Future research should explore the perceptions, views and experiences of staff regarding barriers and facilitators to health promotion in the urgent and emergency care setting, alongside an assessment of patient acceptability.
